# Identification of hand-foot syndrome from cancer patients’ blog posts: BERT-based deep-learning approach to detect potential adverse drug reaction symptoms

**DOI:** 10.1371/journal.pone.0267901

**Published:** 2022-05-04

**Authors:** Satoshi Nishioka, Tomomi Watanabe, Masaki Asano, Tatsunori Yamamoto, Kazuyoshi Kawakami, Shuntaro Yada, Eiji Aramaki, Hiroshi Yajima, Hayato Kizaki, Satoko Hori

**Affiliations:** 1 Keio University Faculty of Pharmacy, Division of Drug Informatics, Tokyo, Japan; 2 Department of Pharmacy, Cancer Institute Hospital, Japanese Foundation for Cancer Research, Tokyo, Japan; 3 Nara Institute of Science and Technology, Nara, Japan; 4 Mediaid Corporation, Tokyo, Japan; National Chiao Tung University College of Biological Science and Technology, TAIWAN

## Abstract

Early detection and management of adverse drug reactions (ADRs) is crucial for improving patients’ quality of life. Hand-foot syndrome (HFS) is one of the most problematic ADRs for cancer patients. Recently, an increasing number of patients post their daily experiences to internet community, for example in blogs, where potential ADR signals not captured through routine clinic visits can be described. Therefore, this study aimed to identify patients with potential ADRs, focusing on HFS, from internet blogs by using natural language processing (NLP) deep-learning methods. From 10,646 blog posts, written in Japanese by cancer patients, 149 HFS-positive sentences were extracted after pre-processing, annotation and scrutiny by a certified oncology pharmacist. The HFS-positive sentences described not only HFS typical expressions like “pain" or “spoon nail”, but also patient-derived unique expressions like onomatopoeic ones. The dataset was divided at a 4 to 1 ratio and used to train and evaluate three NLP deep-learning models: long short-term memory (LSTM), bidirectional LSTM and bidirectional encoder representations from transformers (BERT). The BERT model gave the best performance with precision 0.63, recall 0.82 and f_1_ score 0.71 in the HFS user identification task. Our results demonstrate that this NLP deep-learning model can successfully identify patients with potential HFS from blog posts, where patients’ real wordings on symptoms or impacts on their daily lives are described. Thus, it should be feasible to utilize patient-generated text data to improve ADR management for individual patients.

## Introduction

The incidence of cancers is rising worldwide, and the resulting clinical and economic burden is substantial [1]. Management of adverse drug reactions (ADRs) during cancer treatment directly influences patient compliance, and so can have a crucial influence on the outcome of anticancer treatment [2]. Therefore, early detection of the onset of adverse reactions to anticancer drugs and preventive actions to ameliorate them are important [3–6].

Hand-foot syndrome (HFS), which is also known as palmar-plantar erythrodysesthesia syndrome, is a typical ADR with no curative therapy, and supportive care from an early stage is essential to manage HFS symptoms well [3,7]. HFS tends to occur during chemotherapy with fluoropyrimidine or multi-kinase inhibitors [3,8], and is problematic because it negatively impacts on patients’ quality of life (QOL) and may lead to dose reduction or discontinuation of anticancer drugs [4,7,9–13]. It is common to consider medical intervention for HFS events of Grade 2 or higher per Common Terminology Criteria for Adverse Events published by National Cancer Institute (NCI-CTCAE) ver.5.0 [14]. As guided in the document, Grade evaluation depends on how the HFS events obstruct patients’ activities of daily living (ADL). To accurately understand the impact of HFS events on their ADL, healthcare professionals need to listen intently to patients’ stories or contexts, not only their straightforward complaints. Communication with patients is also key to detecting HFS, as the onset signal relies on each patient’s subjective symptoms. However, there are concerns that HFS symptom signals may be missed in routine medical examinations by physicians, due to the patients’ hesitation to report HFS symptoms during a limited consultation time and their feeling that their cancer treatment should be prioritized [15].

ADR complaints that are not directly reported to healthcare professionals during clinical visits are sometimes recorded at an early stage by the internet patient community [16]. Thus, we hypothesized that it would be possible to capture early ADR signals from patient community data that have not been utilized for medical purposes in order to deliver appropriate care to patients as early as possible. Indeed, some studies have already indicated that internet social communities can be useful for early detection of patient safety issues [17–19]. This trend of early adverse event reporting is consistent with other reports showing that patient-reported adverse events were more frequent and occurred earlier than physician’s assessments [15,20,21] Thus, early detection of adverse events based on patient complaints in the internet community could be a promising approach to manage ADRs more proactively.

In recent years, natural language processing (NLP) deep learning technology has been increasingly applied to various kinds of documents, not only massive data sources, but also text data that requires contextual interpretations [22,23]. Thus, we adopted the deep learning method here to efficiently extract information from our clinically related text data. This technology has already been employed in medical applications, for example, to extract key information from electronic medical records [24–29]. It has been also investigated for patient-generated texts. For example, some studies have been conducted to examine how to utilize NLP-extracted ADRs from patient-authored text data for post-marketing surveillance [30–34]. In addition, technical improvement has been aimed with a competitive shared task in the Social Media Mining for Health Applications (SMM4H) workshop, where Twitter is the main target source to classify or extract tweets with adverse events from those containing drug mentions [35–38]. A prior study on early detection of skin-related ADR from a social health network has been reported [16], but no study has yet been performed to extract more specific definitions, HFS signals, from patient-generated texts. Furthermore, there has been no study of the utility of patient narrative expressions to extract ADR signals in the absence of causative drug information.

Therefore, the purpose of this study was to examine whether cutting-edge deep-learning methods can identify patients with potential ADRs from patient blogs, focusing on HFS.

## Materials and methods

### Overview

**Fig 1:**
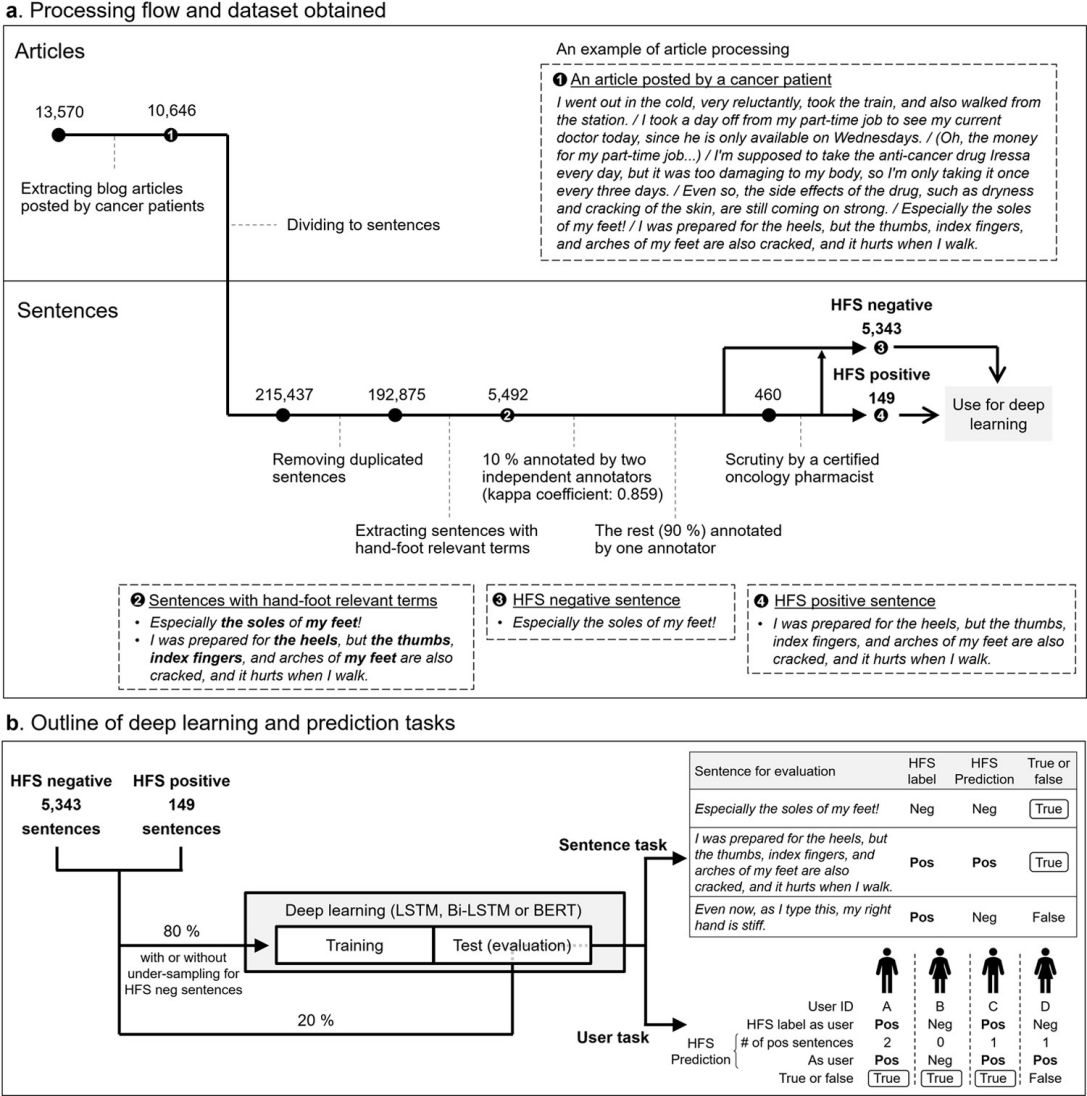
Overview of data processing and deep learning.

This study consisted of two parts; one is article processing, including HFS annotation and scrutiny, and the other is deep learning followed by prediction tasks for evaluation (Fig 1). In the first part, individual blog articles were divided into sentences, and sorted into HFS-positive or negative categories through manual selection by experts to obtain training and evaluation datasets for deep learning (Fig 1A). In the second part, several deep-learning models were trained, and then evaluated using two types of prediction tasks (Fig 1B). Details of each step are described below.

### Pre-processing

Blog articles written in Japanese in the patient web community, Life Palette [39], were utilized for this study. Life Palette is one of the most active internet patient communities in Japan, and many cancer patients post on it. The data source consisted of 13,570 articles written by 289 users, including non-cancer patients, posted on Life Palette from Mar 6^th^, 2008, to Nov 20^th^, 2014.

First, 10,646 articles were extracted as blog articles written by cancer patients, which were a total of 207 users. Afterwards, all the articles were divided into sentences by using the publicly available open-source “ja_sentence_segmenter” [40]. This afforded 215,437 sentences. Duplicated sentences were removed, majority of which were just including punctuation marks or a few words, leaving 192,875 independent sentences (Fig 1A).

These sentences were processed to extract those that contained hand-foot-relevant terms, such as “hand”, “foot”, “finger”, and “nail”, based on the assumption that HFS signal sentences should include at least one hand-foot-related word (Fig 1A). This pre-processing step was introduced to increase the likelihood of including potential HFS expressions and also save the cost of following annotation step.

### HFS-positive and negative sentences

An annotation guideline for HFS was created to conduct annotation in a unified way among several annotators, referring to the HFS management literature [3,7,9–12] (Table 1). Specific keywords were used to annotate HFS-like expressions (e.g. “pain”, “swelling”, “paresthesia”, and “deformed nail”). In addition to these pre-determined key words, patient-derived unique expressions were also annotated as HFS if they appeared to refer to HFS-like symptoms. This annotation guideline was aimed to broadly pick up HFS-like expressions, including undefined terms like those patient-derived unique ones, to convey all potential HFS expressions to the final scrutiny step by a certified oncology pharmacist. The scrutiny step is detailed in the two paragraphs below of this section.

**Table 1:** Annotation guideline.

Definition	Hand-foot syndrome-like symptoms possibly caused by anti-cancer drug
Positive criteria	Including at least one term listed below, being described with hand-foot-relevant terms. • pain • numbness • swelling • flushing • eczema • feeling of wrongness • paresthesia / dysesthesia • deformed nail • other patient-derived unique expressions possibly indicating HFS
Exclusion criteria	• Symptoms not on-going (i.e., already recovered, possibility for the future, description of general symptoms, reference to other source articles, imagination) • Unidentified complaint (i.e., weary, listless, etc.) • Existing clear causality other than drug (i.e., walking, blood sampling, etc.) • Swelling caused by water retention

Using this annotation guideline, two researchers (SN, MA) separately conducted annotations for 10% of the population with hand-foot-relevant terms. We confirmed that the results of the two annotations showed a high kappa coefficient, [41,42] indicating a high degree of concordance. Then, one of the annotators (SN) completed the HFS annotation for the rest of the population (Fig 1A).

Following the annotation process, a Japanese Society of Pharmaceutical Health Care and Science-certified oncology pharmacist (KK), who regularly sees and advises cancer patients at a dedicated cancer hospital, further evaluated the annotation results to differentiate likely and unlikely expressions of HFS (Fig 1A). This scrutiny process was a crucial step in this study to bring real-world pharmacological and clinical expertise into the selection of expressions as HFS-positive data.

The HFS-positive sentences after the scrutiny by a certified oncology were then divided at a 4 to 1 ratio to obtain training and evaluation datasets. For HFS-negative data, all or a part of the remaining sentences were used (Fig 1B). Specifically, for the test step, randomly selected HFS-negative sentences were added to HFS-positive sentences to obtain an evaluation dataset with the same HFS proportion as the original population. On the other hand, for the training step, three patterns of HFS-negative sentences were added to training datasets in the ways described in detail in the results section.

### Deep-learning method

NLP models utilized for this study were long short-term memory (LSTM), bidirectional LSTM (Bi-LSTM) and BERT [43–46], which are state-of-the-art deep-learning methods to analyze the context of language. To efficiently learn Japanese word embeddings, we utilized publicly available trained datasets in Japanese for pre-training: “fastText”, which is open pre-trained dataset based on Japanese Wikipedia articles provided by Facebook, for LSTM and Bi-LSTM, and a pre-trained Japanese BERT model released by Tohoku University for BERT [47,48]. As tokenizers, Janome and MeCab were utilized for LSTM or Bi-LSTM, and BERT, respectively [49,50]. The training was performed with a 5-fold cross-validation method for each model.

### Task and evaluation parameters

Two prediction tasks were set for evaluation; one is to predict HFS-positive or negative for individual sentences (the “sentence task”), and the other is to differentiate HFS-positive patients or negative patients (the “user task”) (Fig 1B). The user task aims to prioritize identifying patients themselves, rather than individual sentences, with the ultimate goal of issuing alerts or supportive information to possible HFS patients. We evaluated the performance of the trained models in these two tasks in terms of precision, recall and f_1_ score.


$$Precision=\frac{\#of\;True\;Positive}{\#of\;True\;Positive+False\;Positive}$$

$$Recall=\frac{\#of\;True\;Positive}{\#of\;True\;Positive+False\;Negative}$$

$$F_{1}\;score=\frac{\;2*precision*recall}{precision+recall}$$

### Ethical considerations

In accordance with Life Palette’s terms of service, agreements from all contributing users for secondary anonymous use of blog posts by a third party for research purposes were obtained on their individual start dates of service use, by checking the Agree button on the website. Our laboratory (Keio University) obtained blog post data for research purpose from the operating company, Mediaid Corporation, based on a joint research agreement. This study was conducted with their anonymized data, following approval by the ethics committee of the Keio University Faculty of Pharmacy (approval No. 190301–1). Informed consent specific for this study was waived due to the retrospective observational design of the study.

## Results

### Dataset

The pre-processing yielded 5,492 sentences with at least one hand-food-related wording (Fig 1A). The mean and the median (Min-Max) number of words in the 5,492 sentences were 68.80 and 44 (3–1328) words, respectively.

Potential HFS-positive sentences among the 5,492 sentences were annotated by two independent annotators, with reference to the annotation guideline (Table 1). When the two annotators had annotated 10% of the population, the kappa coefficient was 0.859, indicating a high degree of consistency between the results of the two separate annotations. Finally, 460 potential HFS-positive sentences were obtained from this annotation process (Fig 1A).

Afterwards, the certified oncology pharmacist reviewed the 460 potential HFS-positive sentences, and extracted ones that appeared valid based on clinical experience. This scrutiny process left 149 sentences that were finally identified as HFS-positive expressions (Fig 1A), corresponding to approximately 2.7% of 5,492 sentences with hand-foot-relevant terms.

### HFS-positive users/sentences

Some examples of the 149 HFS-positive sentences are shown in Table 2. These include not only expressions typical of HFS, such as “pain/painful” or “spoon nail”, but also patient-derived onomatopoeic expressions, such as “The skin of my hand is peeling off like BERO-BERO (seriously)” or “BUYO-BUYO (something like blisters) on my feet”. The mean and the median lengths of the 149 HFS-positive sentences (Min-Max) were 52.06 and 37 (11–235) words, respectively (Table 3).

**Table 2:** Examples of HFS positive sentences.

Japanese	English (translated for reference)
ところが、4月後半になると階段を上るときに足に痛みを感じるようになった。	However, in the latter half of April, I started to feel pain in my legs when I climbed the stairs.
どうも僕の指の爪は、スプーン爪と呼ばれる状態になっているらしい。	Apparently my fingernails are in a state called spoon nails.
ちょっと爪先がどこかに当たっただけではがれて2枚爪になったりするし、ポロポロと欠けることも多い。	If the tip of my nail hits somewhere, it will come off and split into two, and it will often be chipped.
朝から、手のひら、足裏が真っ赤。	From the morning, the palms and soles are bright red.
手の皮がべろべろに剥けている。	The skin of my hands is peeling off like BERO-BERO (seriously).
足のブヨブヨは、すっかり固くなってる感じ。	The BUYO-BUYO (something like blisters) on my feet is becoming completely stiff.

**Table 3:** Statistical parameters for HFS-positive sentences.

# of HFS-positive sentences (sentences)	149
Length of HFS-positive sentences (words)	
• Mean	52.06
• Mean (Min–Max)	37 (11–235)
# of blog posts that included HFS-positive sentences (articles) … [*1]	110
• Out of which [*1], # of blog posts that also included specific anti-cancer drug names (articles) … [*2]	25
• Out of which [*2], # of blog posts that described timing of starting the anti-cancer drug (articles)	4
# of patients who posted HFS-positive blog posts (people)	42
# of posted HFS sentences per patient (sentences)	
• Mean	3.55
• Mean (Min–Max)	2 (1–24)

The number of blog posts that included HFS-positive sentences was 110. Among the 110 articles, 25 included specific anti-cancer drug names within the blog post, based on our manual review, but among them, only 4 articles described how long the patient had taken the anti-cancer drug (Table 3). There was no comprehensive information regarding prior or concomitant drugs in the blog posts, and no medical diagnosis except for the primary disease of cancer.

The number of users who posted HFS-positive blog posts was 42. The mean and the median numbers of posted HFS sentences per user (Min-Max) were 3.55 and 2 (1–24) sentences, respectively. It was confirmed that a single user tends to post multiple HFS-related sentences in blog articles (Table 3).

### Training and performance evaluation

For training, three datasets were prepared to evaluate the performance scores for the two predefined tasks: sentence task and user task. The first training dataset included the same proportion of HFS as the original population and the evaluation dataset, i.e., approximately 2.7% (designated as “Original”). Secondly, an under-sampling method was tried to address imbalance in the training dataset, which could potentially cause over-fitting to majority data. To evaluate different patterns of under-sampling, two training datasets were prepared: one with a 1-to-1 ratio of HFS-positive and negative sentences, and the other with a 1-to-20 ratio of HFS-positive and negative sentences (designated as “Balanced” and “Under-sampling” respectively).

The performance scores are listed in Table 4. The best f_1_ score for the sentence task was 0.54 in BERT with “Under-sampling” (Table 4A). For the user task, the best f_1_ score was 0.71 in BERT with “Under-sampling” (Table 4B). Training with “Balanced” improved recall dramatically, especially in LSTM or Bi-LSTM for both tasks, but negatively impacted on precision, resulting in relatively low f_1_ scores (Table 4A and 4B).

**Table 4:** Performance score.

a. Sentence task			
	Precision	Recall	f_1_ score
LSTM			
Original	0.28	0.20	0.23
Balanced	0.10	0.96	0.19
Under-sampling	0.41	0.33	0.37
Bi-LSTM			
Original	0.35	0.33	0.34
Balanced	0.15	0.86	0.26
Under-sampling	0.33	0.46	0.38
BERT			
Original	0.43	0.23	0.30
Balanced	0.03	0.56	0.07
Under-sampling	0.45	0.66	0.54
b. User task			
	Precision	Recall	f_1_ score
LSTM			
Original	0.66	0.52	0.58
Balanced	0.23	1.00	0.37
Under-sampling	0.57	0.57	0.57
Bi-LSTM			
Original	0.65	0.68	0.66
Balanced	0.30	1.00	0.46
Under-sampling	0.50	0.68	0.57
BERT			
Original	0.53	0.36	0.43
Balanced	0.13	0.93	0.23
Under-sampling	0.63	0.82	0.71

Precision, recall and f_1_ scores are shown in these tables for the sentence task (a) and the user task (b). NLP deep-learning models used for this study are LSTM, Bi-LSTM and BERT. The percentage of positive data in the training dataset is approx. 2.7% for “Original” (the same ratio as in the original population), 50% for “Balanced” and 5% for “Under-sampling”.

## Discussion

Our results indicate that cutting-edge NLP technologies can be utilized for the identification of potential HFS patients from blog articles, confirming that it is feasible to extract patients with HFS from patient-generated text data by using NLP deep learning. It was also demonstrated that patient-derived unique HFS expressions, such as onomatopoeic expressions, can be correctly learned and extracted from patients’ blogs by NLP deep-learning models. Importantly, our approach can extract potential ADR signals from patient-generated information on the web without relying on causative drug information, suggesting that this could be versatile approach for the early detection.

In our study, we utilized three NLP deep-learning models, LSTM, Bi-LSTM and BERT, for extraction of HFS-positive sentences. LSTM and Bi-LSTM are recurrent neural network models that have been under development since 1997 [43–45], whereas BERT emerged in 2018 [46]. In previous studies to extract ADR information from Twitter or health-related social media, the f_1_ scores were around 0.5 to 0.7 [31,32,34]. The range of f_1_ score was also comparable in the SMM4H shared tasks in 2020 or 2021, where some cutting-edge BERT based systems were exploratorily employed [35–38]. Another application of an NLP model for early detection of skin-related ADR from a social health network achieved a micro-averaged f score of 0.74 [16]. In our study, the best f_1_ score in the sentence task was 0.54 in BERT with “Under-sampling” (Table 4A). Although the performance may not seem high compared with previous research described above, it should be considered that our data source does not necessarily contain causative drug information within the sentences, which leads to a lower density of ADR mentions than in the case of sentences with causative drug information. Further adjusting the training set or learning parameters can improve the performance scores in the sentence task, and this might greatly improve the detection of individual ADR mentions from patient blogs. On the other hand, the best f_1_ score in the user task was 0.71 in our study (Table 4B), which is comparable to those in previous research [16,31,32,34–38], although it should be highlighted that our model had a narrow focus on HFS among ADRs, unlike previous studies. The ability of the deep-learning method to identify expressions characteristically used by patients to describe HFS was confirmed by a certified oncology pharmacist, who had abundant relevant clinical experience. The HFS-positive patient blogs tended to have multiple HFS-positive sentences in one article (Table 2), and this would be the reason why the deep-learning model showed consistently better performance scores in the user task than in the sentence task. When prioritizing identification of patients with ADR signals in a future implementation phase, the performance scores in the user task should be strongly emphasized. The f_1_ scores in the user task suggest that BERT is the best deep-learning tool to automatically identify potential HFS patients from patient blogs, though the performance scores of individual models might be improved by adjusting the training dataset, conditions or system itself, as tried using SMM4H [35,36]. Performance in the sentence task might be similarly improved.

One of the goals of our research is to utilize patient-generated texts and deep learning analysis for early detection of patients with ADR signals. As patient blog posts are not grammatically systematized and have relatively large word counts per sentence (the average was 52.06 words per HFS-positive sentence [Table 3]), this is not easy data source to handle, and the deep learning method is thus the preferred method, even though the scale of the data source itself was not large (the number of HFS-positive sentences was just 149 among 5,492 [Table 3]). In terms of early detection of ADR signals, we looked into two particular cases which included both a specific anti-cancer drug name and the timing of starting the drugs in their blog posts. One mentioned that “It has been 9 weeks since I started to take erlotinib”, and a complaint of “Cracked fingertips are stressful when working in the kitchen” was mentioned. The other said “On the 79th tablet from the start of gefitinib” and “The skin symptom has emerged on my hands and feet, but as it is not itchy and so bad, I don’t use any medications for now”. Based on published information, HFS caused by kinase inhibitors will commonly occur within first 12 weeks from the start of medication [3,11,13], so it cannot be concluded that the 9th week or the 79th tablet (day) from the start truly represents early detection. As most of the patient blogs in this study did not name a specific anti-cancer drug name or state the timing of starting the drugs (Table 4), it was infeasible to assess the sensitivity of these models for early detection of ADR signals in this study. To further investigate this aspect, prospective studies need to be conducted with records of comprehensive time-course information, including the start dates of individual anti-cancer drugs, dates of clinic visits, and medical diagnosis of any ADRs, etc., in addition to patient-generated daily texts.

False-positive analysis was also conducted to assess the reliability of the models. We found that some sentences which the NLP models predicted as “positive” were closely similar to positive sentences (for example, such false-positives included “Light numbness at the tip of the finger” or “I have rash on my right lower leg”). They were also included in “potential HFS-positive” sentences annotated by the two researchers (Fig 1A). This may mean that the deep-learning models constructed for HFS detection could be useful for broader detection of adverse events related to the skin.

In clinical practice, it is crucial to distinguish causative drugs, because the clinical course may differ depending on the administered drug; for example, strictly speaking, the symptoms caused by fluoropyrimidine and by multi-kinase inhibitors are designated differently as “hand-foot syndrome” and “hand-foot skin reaction”, respectively [8]. In examining the HFS-positive sentences in our dataset, we found that those two symptoms may be described with different expressions by patients (for example, “cracked fingertips” was a typical expression associated with HFS caused by fluoropyrimidine). Thus, it may be possible to identify causative drugs using the present deep-learning systems. Another possibility is severity assessment based on patient-generated texts. Some HFS-positive sentences indicated how the symptoms affected patients’ daily lives. These statements can be interpreted as Grade 2 or 3 according to NCI-CTCAE ver.5.0 [14], and thus deep-learning models might also be applicable to assess severity. These targets might require larger training datasets, as a limitation in our study was the relatively small dataset used for deep learning. Larger training datasets might also improve the performance scores for HFS identification. In the future, it would be desirable to incorporate data from other sources, such as Twitter or other internet community forums, or to collect additional data from cancer patients through interviews or prospective studies.

This system might be extended in various ways. One possibility would be to examine a much wider range of patients’ expressions, including those referring to ADRs other than HFS. This should be feasible, since we confirmed that our deep-learning models can identify distinctive wordings for HFS, including unique patient-derived onomatopoeic expressions, even from “noisy” sentences with an average of 52.06 words (Table 3). Another possibility, given that this system can identify HFS patients from just a single sentence, would be to apply this system to a wider range of source data, including short text sources such as Twitter. Although it is expected that the deep-learning method would show optimum performance when reading out context from relatively complicated sentences, like the data source of this study, the models constructed from patient blogs may be applicable to a broader range of patient text data.

Incorporation of the system developed here into internet patient communities could be useful to automatically issue an alert to patients with potential ADR, encouraging them to visit the clinic earlier than planned. The system could also provide patients with self-management measures via the web to prevent further symptom deterioration. It also seems likely that introduction of these novel features into internet communities would encourage patient themselves to proactively record their disease experiences. If the performance of f_1_ score 0.71 proves to be insufficient for practical implementation, it would be possible to incorporate follow-up questions for patients in order to differentiate HFS and the other symptoms more accurately, for example to clarify the location of adverse events on the body.

In conclusion, we suggest that our NLP deep-learning system to identify users with HFS from their internet blog posts has the potential to improve individual patients’ ADR management.
